# A preliminary investigation towards automated computation of Multiparametric strain Z-Score in dilated cardiomyopathy using navigator-gated spiral DENSE MRI and radial point interpolation method

**DOI:** 10.1186/1532-429X-18-S1-W11

**Published:** 2016-01-27

**Authors:** Julia Kar, Brian P Cupps, Danielle Koerner, Kevin Kulshrestha, Michael K Pasque

**Affiliations:** Washington University, St. Louis, MO USA

## Background

To investigate the effectiveness of a rapid, clinically viable Multiparametric strain Z-Scores (MPZS) risk stratification metric for identifying sentinel regions of myocardial dysfunction in non-valvular, non-ischemic, dilated cardiomyopathy (DCM). The MPZS metric emphasizes relative severity of myocardial injury in sentinel sub-regions of the left ventricle (LV).

## Methods

Multi-observer analysis was conducted by imaging DCM patients (N = 15) using the 3D Navigator-gated Spiral Displacement ENcoding with Stimulated Echoes (DENSE) MRI. Automated segmentation of the myocardium was conducted with the MASS (Medis) software which was followed by dividing the left-ventricle into 16 AHA recommended sub-regions (sans apical cap). Meshfree Radial Point Interpolation Method (RPIM) numerical analysis was used for 3D Lagrangian strain computation. Dimensionless MPZS was computed by linearly combining z-scores of three 3D normal strains (radial, circumferential and longitudinal) which were first normalized with strains from a healthy subjects' database (N = 40). Identification of the septal region as the sentinel region of highest dysfunction was conducted using MPZS. Color coded surface maps of MPZS was generated for the full epicardium and endocardium. The time taken for analysis from start of scan to MPZS computation was recorded for each patient.

## Results

Computations of sub-regional MPZS showed highest contrast between basal posterolateral and anteroseptal sub-regions (1.8 ± 0.3 versus 0.7 ± 0.4) and mid-wall posterolateral and anteroseptal sub-regions (1.9 ± 0.6 versus 0.8 ± 0.5) in the LV myocardium. The highest MPZS recorded in a patient were 2.1 in the basal posterolateral and 2.2 in the mid-wall posterolateral sub-regions. The time taken for the navigator-gated DENSE scan was 18 ± 5 minutes, the time taken for automated segmentation was approximately 15 minutes and the RPIM based MPZS computations took 2 ± 0.9 minutes per patient. In comparison, traditional MRI sequences require more than an hour for scanning and at least a full workday for post-processing.

## Conclusions

Our technique shows that LV contractile dysfunction can be analyzed in an automated manner and show the (historically accurate) higher septal contractile dysfunction over lateral sub-regions. Our methodology also show that diagnosis of heterogeneous dysfunction in the myocardium can be streamlined by a factor of several hours over traditional methods.

**Figure 1:**
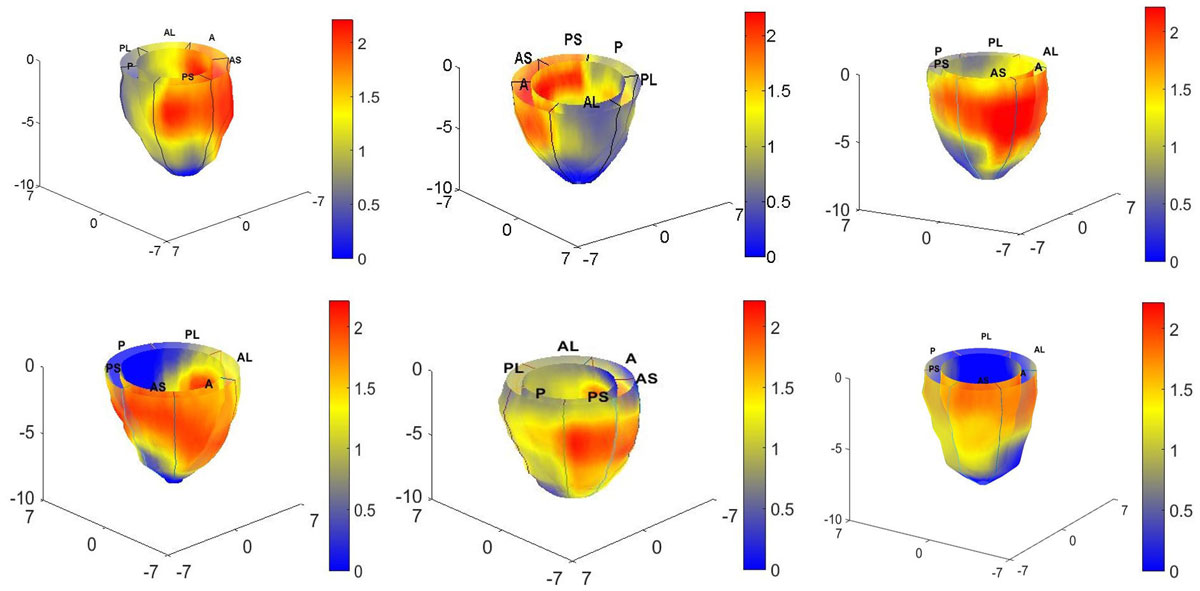
**Maltiparametric strain Z-Scores distributionin in the epicardiums and endocardiums of six of the 15 non-Ischemic, dilated cardiomyopathy patients**.

